# Huddling and food availability shape seasonal torpor and energetics of juvenile garden dormice

**DOI:** 10.1016/j.isci.2026.116211

**Published:** 2026-06-04

**Authors:** Laura Magaly Charlanne, Beata Sente, Sebastian G. Vetter, Joy Einwaller, Johanna Painer-Gigler, Audrey Bergouignan, Alexandre Zahariev, Caroline Gilbert, Sylvain Giroud

**Affiliations:** 1 Université de Strasbourg, CNRS, IPHC DEPE UMR 7178, Strasbourg F-67000, France; 2 Research Institute of Wildlife Ecology, Department of Interdisciplinary Life Sciences, University of Veterinary Medicine Vienna, Vienna, Austria; 3 Veterinary Public Health and Epidemiology, Department for Farm Animals and Food System Science, University of Veterinary Medicine Vienna, Vienna, Austria; 4 UMR 7179, CNRS/MNHN, École Nationale Vétérinaire d’Alfort, Laboratoire MECADEV, Maisons-Alfort, France; 5 Energetics Lab, Department of Biology, Northern Michigan University, Marquette, MI, USA

**Keywords:** animal physiology, biological sciences, rodent behavior

## Abstract

Environmental fluctuations challenge juvenile hibernators to efficiently balance growth, fattening, and energy conservation prior to their first winter. This study investigates how social thermoregulation (huddling) and food availability affect the use of torpor, growth, fat accumulation, and hibernation in late-born juvenile garden dormice. Juveniles exhibited flexible energy-saving strategies, with huddling influencing torpor patterns and food intake during pre-hibernation. Despite these behavioral differences, individuals reached similar body mass and size at the onset and end of hibernation, regardless of housing or feeding conditions. During hibernation, food availability modulated total energy expenditure, particularly in males, likely due to changes in reproductive investment. These findings suggest that the combined use of torpor and huddling provides developmental flexibility, enabling juvenile dormice to meet the energetic demands of early life. This plasticity may be critical for survival in the face of environmental unpredictability, and future studies should investigate how early-life energy strategies influence long-term fitness and reproduction success.

## Highlights

• Huddling modulates torpor patterns and food intake in juvenile dormice
• Huddled vs. single juveniles reach similar body mass before and after hibernation
• Males are more impacted by food availability than females in hibernation behavior
• Torpor and huddling enable flexible responses to environmental variability

## Graphical abstract

**Figure:**
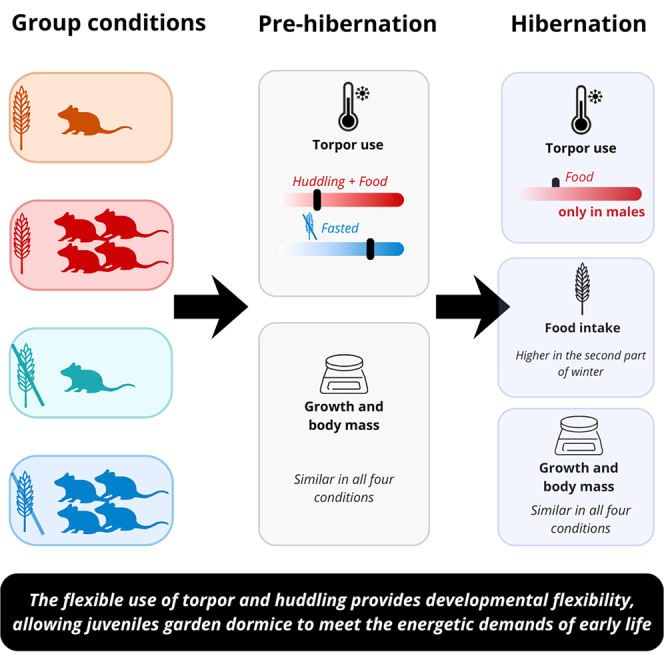

## Introduction

Environmental variability is a fundamental selective force shaping the evolution of flexible physiological and behavioral responses of organisms. Ongoing global change is expected to increase the frequency and unpredictability of extreme climatic events, thereby intensifying these challenges. In seasonal environments, animals often face periods of food shortage and must employ adaptive strategies to optimize the allocation of limited resources.^1^ Energy-saving strategies are particularly important for small-bodied species, whose high surface-to-volume ratio leads to greater heat loss. Such strategies include food storage, migration (e.g., in birds), social thermoregulation (i.e., huddling), and active reductions in metabolic rate and body temperature (T_b_), such as daily torpor and hibernation in birds and mammals.^2^^,^^3^^,^^4^

Torpor is an energy-saving strategy used by a wide range of bird and mammal species living in climatic regions ranging from arctic and alpine areas to the tropics.^4^ During times of cold, food shortage, or drought, animals drastically reduce their metabolic rate in an active and controlled manner, inducing a lowering of their T_b_.^5^^,^^6^ Based on the duration of the hypometabolic state, two different forms of torpor can be distinguished: daily torpor and hibernation. Daily torpor is characterized by torpor episodes lasting less than 24 h, allowing animals to continue foraging during the rest of the day.^7^ On the other hand, hibernation is defined as a succession of torpor bouts lasting longer than 24 h, usually days to several weeks with a drastic reduction of metabolic rate (until ∼4% of the basal rate) and a minimum T_b_ of ∼2°C–6°C.^4^ During hibernation, the state of torpor is regularly interrupted by phases of rewarming, called periodic arousals that usually last less than 14 h. During periodic arousals, animal’s metabolic rate increases drastically to allow the body to reach euthermic T_b_ values around 36°C–37°C in small mammals. Yet, periodic arousals are very costly and account for most (70%–80%) of the energy demand during the hibernation season.^8^^,^^9^^,^^10^

Another powerful energy-saving strategy is the use of social thermoregulation or “huddling.” To date, social thermoregulation has been observed in more than 67 mammal species, including the garden dormouse (*Eliomys quercinus*), and 25 bird species.^3^ Huddling can be defined as a cooperative group behavior where animals aggregate closely together to minimize heat loss and lower energy expenditure by 6%–53%,^3^ which is especially important for juveniles, due to their small body size, as illustrated by energy savings realized via huddling in rabbit pups.^11^ One of the main advantages of huddling is the ability to be combined with other behaviors. Heterotherms may employ the strategy of huddling in association with the use of torpor to minimize the energetic costs induced by the periodic arousals.^12^^,^^13^

Prior to winter, it is crucial for hibernators to accumulate sufficient energy reserves, as most hibernating species do not eat and rely entirely on their body fat reserves during hibernation.^14^ Hence, the level of pre-hibernal fat stores is a good predictor of winter survival.^14^ However, the efficacy of hibernation in buffering body mass loss and ensuring winter survival highly depends on the extent of metabolic depression, which is constrained by the temperature within the hibernacula in which individuals spend the winter. Hence, warmer temperatures are expected to greatly affect the winter survival of hibernating species in the context of ever-increasing global warming and environmental fluctuations.

During late summer and fall, juveniles from hibernating species face an additional challenge: allocating energy for growth and accumulating sufficient fat reserves to survive winter hibernation. Hence, both processes of growth and fat accumulation are crucial and must occur within a short time period (several weeks), especially in juveniles from a second litter that are born late in the reproductive season, as they may already face reduced food availability and lowered ambient temperatures.^11^^,^^15^ Previous studies show juvenile use torpor to save energy and gain fat depots, though it is not compatible with growth, as both metabolic rate and T_b_ are decreased, optimal growth requiring a warm body to occur.^15^^,^^16^^,^^17^ Consequently, the combination of different energy-saving strategies could confer sufficient flexibility to young hibernating species, enabling them to cope with early-life challenges, especially in the context of global change.

The garden dormouse (*Eliomys quercinus*), a small European hibernating rodent, is known to use both huddling and torpor strategies.^3^^,^^18^^,^^19^ This hibernator usually does not store food and relies entirely on body fat reserves, but can forage during mild winters when food is available.^20^ During the active season, individuals may produce up to two litters before accumulating fat reserves to prepare for hibernation. Juveniles from the second litter therefore have fewer time to develop and prepare for winter relative to earlier-born conspecifics. In the wild, garden dormice generally start hibernation in autumn (September–October) and remain in the hibernacula until spring (March–April), depending on environmental conditions. Body mass varies seasonally, ranging from approximately 40–50 g in juveniles at weaning to substantially greater body masses (90–110 g) after pre-hibernation fattening at hibernation onset, reflecting the strong reliance on endogenous fat reserves during winter.^17^ A recent study on juvenile garden dormice reported no energetic benefit of huddling, irrespective of food availability, over an entire winter at the individual level.^9^ The same study, however, revealed a significant effect of huddling on the energetics of the torpor-arousal cycle as it reduced heat exchange and mass loss by two-thirds in huddled compared to single individuals during rewarming, hence asking for further investigations during the various phases of hibernation. Juvenile garden dormice also rely on a flexible use of torpor during development, which is low in the first weeks of intensive growth but increases in the late phase of pre-hibernation fattening.^17^ This study demonstrated that IF garden dormice used torpor more frequently than individuals fed *ad libitum* (AL), and torpor use of fasted individuals correlated positively with their body (fat) mass gain. Yet, very little is known concerning the energetic benefit of the combined use of torpor and huddling before and during their first winter.

In addition to environmental and energetic constraints, sex-specific life-history strategies may influence hibernation patterns. In many hibernating mammals, males and females differ in the timing of emergence from hibernation, males often emerging earlier than females in order to establish territories and prepare for the upcoming breeding season, as reported in hibernating Arctic ground squirrels (*Spermophilus lateralis*).^16^ Although such patterns are well documented in adults, it remains unclear whether sex-specific differences in activity and torpor use may already arise during the first winter of life, when individuals face strong energetic constraints, but future reproductive success may still favor early preparation for males.

Therefore, this study aims to investigate the effect of huddling on (i) the use of torpor during pre-hibernation and winter hibernation, (ii) the processes of growth and body and fat mass gain prior to hibernation, (iii) the levels of body (fat) content and body size at the hibernation onset, and (iv) subsequent hibernation behavior and body mass loss over winter in late-born male and female juvenile garden dormice according to food availability (“AL” vs. “IF”). Specifically, we predict that (i) huddling will minimize the use of torpor during pre-hibernation and (ii) reduced food availability will increase torpor use, leading to delayed growth. We further predict that huddling will allow juveniles (iii) to foster their development and body mass gain, and to reach similar fat content but higher body size than isolated individuals. Finally, we expect (iv) no substantial energetic benefit of huddling for juveniles during the entire winter, but instead predict, like hibernating squirrels, male juvenile dormice will be more active than females, especially at the end of hibernation, to be able to prepare for subsequent reproduction.

## Results

**Figure 1:**
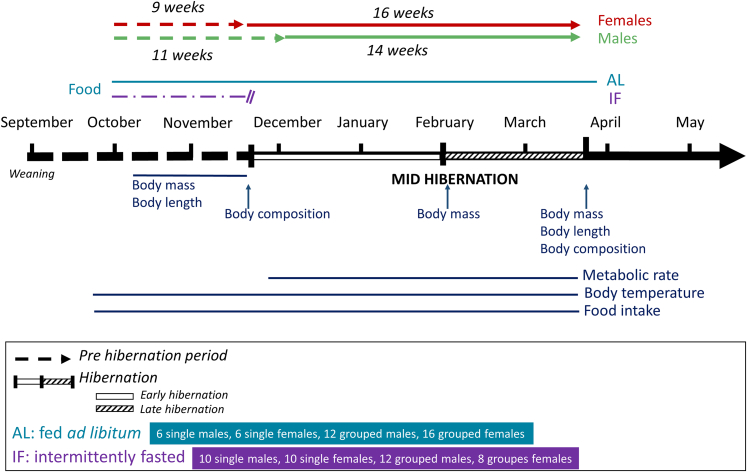
Schematic overview of the experimental design Experiments were conducted over two consecutive years following an identical protocol, except for body composition measurements, which were performed only during the 2019–2020 period. Parameters measured are indicated in blue. Differences between males and females are represented in green and red, respectively.

To test our hypotheses, we designed a factorial experiment reported in Figure 1.

### Pre-hibernal period

The pre-hibernal phase lasted nine weeks for females and eleven weeks for males, as females entered two weeks earlier into hibernation than males (Nov. 20^th^ for females, and Dec. 4^th^ for males).

### Torpor use

**Figure 2:**
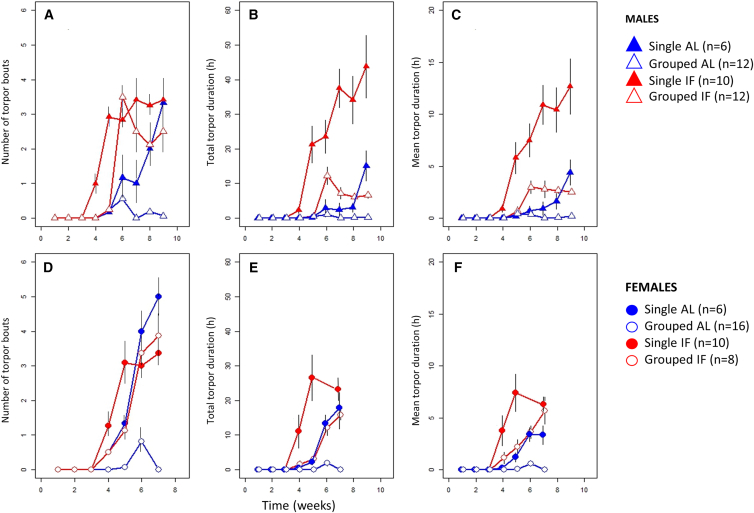
Changes in torpor use during the pre-hibernal period Changes in torpor frequency (A and D), total torpor duration (B and E), and mean torpor duration (C and F) of males and females juvenile garden dormice either fed “AL” or “IF” from the start of experiments until hibernation onset. Torpor patterns were assessed by measurements of nest temperature. Values are means ± SD. *N* = 80.

Torpor frequency differed according to diet, sex, and housing conditions over time (Table 1). In the last phase of pre-hibernation single individuals showed a higher torpor frequency compared to grouped individuals. This effect was higher in fasted individuals than in dormice fed AL. Females started to use torpor earlier and with a higher frequency than males in the last phase of pre-hibernation (Figure 2). Total torpor length also differed according to diet, sex, and housing conditions (Table 1). Single individuals showed a longer total torpor duration shortly before entering winter hibernation than individuals that huddled (Figure 2). Fasted individuals remained torpid longer over the whole period than animals with food AL. Additionally, females started torpor use earlier and exhibited a longer torpor duration than males. However, during the last three weeks of pre-hibernation, the total torpor length in fasted males surpassed that of males fed AL and all females. Finally, single fasted garden dormice spent on average a longer duration torpid than huddling individuals with food, and this effect was stronger in males (Figure 2 and Table 1). Representative individual temperature profiles for each experimental group are shown in Figure S1.

**Table 1:** Parameters of linear models for the effects of housing conditions, sex, diets and time on pre-hibernation torpor parameters of juvenile garden dormice

Response variable	Term	Estimate ±SD	t value	*p* value
Number of torpor bouts	Sex	0.65 ± 0.29	2.19	**0.03**
	Housing conditions	−0.60 ± 0.31	−1.94	**0.05**
	Diet	−0.77 ± 0.30	−2.56	**0.01**
	Time	0.29 ± 0.05	6.13	**<0.001**
	Sex∗time	−0.25 ± 0.05	−5.19	**<0.001**
	Group∗time	0.3 ± 0.05	6.45	**<0.001**
	Diet∗time	0.32 ± 0.05	6.82	**<0.001**
Total torpor length (h)	Sex	1.28 ± 2.88	0.44	0.66
	Housing conditions	−10.31 ± 3.19	−3.23	**0.002**
	Diet	−5.29 ± 3.01	−1.76	0.09
	Time	0.32 ± 0.36	0.87	0.38
	Sex∗time	−0.97 ± 0.37	−2.60	**0.009**
	Group∗time	3.76 ± 0.36	10.50	**<0.001**
	Diet∗time	3.19 ± 0.35	9.02	**<0.001**
Mean torpor length	Sex	0.15 ± 0.84	0.17	0.86
	Housing conditions	−2.72 ± 0.93	−2.92	**0.004**
	Diet	−1.69 ± 0.87	−1.93	0.06
	Time	0.05 ± 0.11	0.49	0.62
	Sex∗time	−0.19 ± 0.11	−1.83	0.07
	Group∗time	0.99 ± 0.10	9.64	**<0.001**
	Diet∗time	1.01 ± 0.10	10.01	**<0.001**

Housing conditions were either single or grouped, and diets were *ad libitum* (AL) or food-restricted (IF). *p* values in bold indicate statistically significant effects.

**Figure 3:**
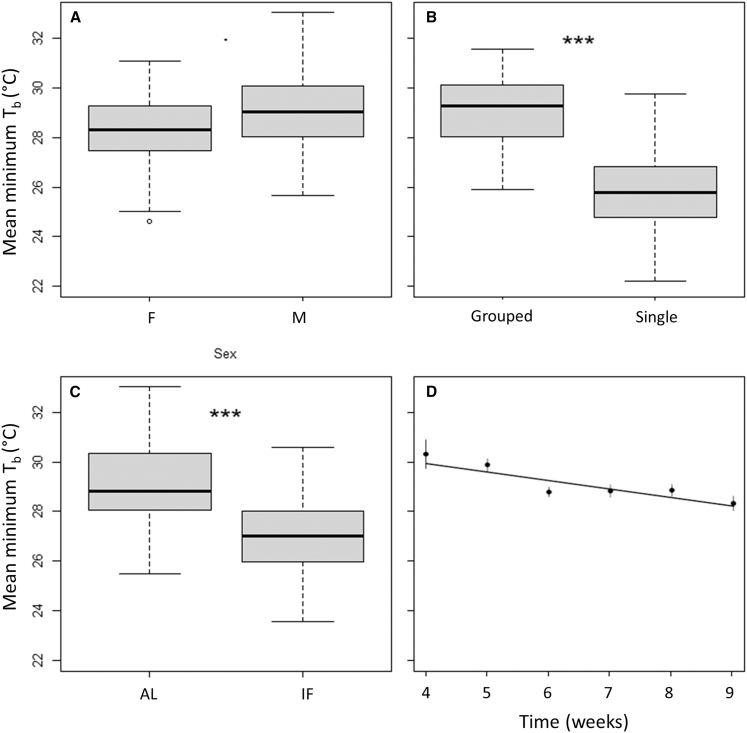
Mean minimum T_b_ during the pre-hibernal period Mean minimum T_b_ (°C) between females (*n* = 40) and males (*n* = 40) (A), different housing conditions (grouped *n* = 48, single *n* = 32, (B), different diet *(ad libitum* = AL *n* = 40, fasted *n* = 40, C), and over time (weeks, D). Values are means ± SD. Significance was assessed using type II ANOVA (Wald χ^2^ tests) on linear mixed-effects models. Asterisks indicate significance levels (∗∗∗*p* < 0.001).

**Figure 4:**
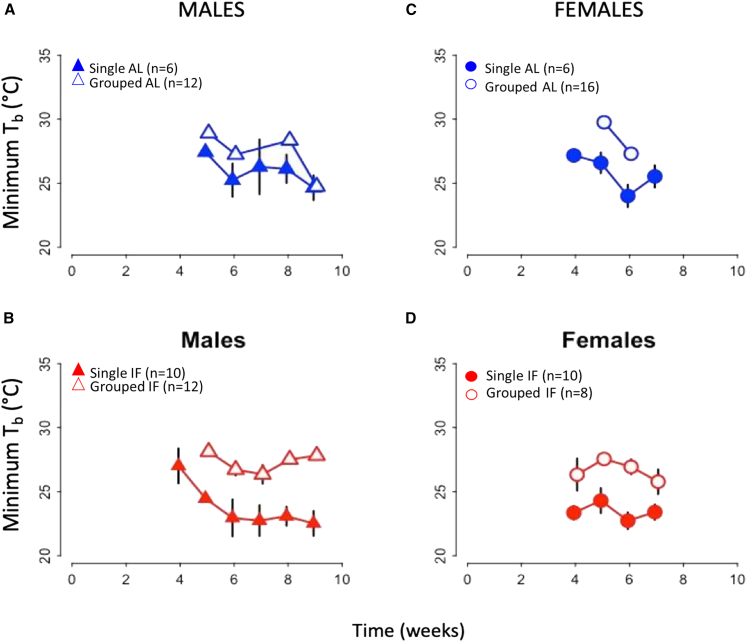
Minimum T_b_ over time during the pre-hibernal period Mean minimum T_b_ (°C) over time (weeks) between males (*n* = 40 A and B) and females (*n* = 40 C and D) in different diet conditions (*ad libitum* = AL *n* = 40, fasted *n* = 40) and housing conditions (grouped *n* = 48, single *n* = 32) from the start of experiments until hibernation onset. Values are means ± SD.

During torpor, the mean minimum T_b_ differed significantly between housing conditions, diets, and time (Table 2). Single individuals and fasted individuals entered deeper torpor compared to huddled garden dormice and individuals kept with food AL (Figures 3 and 4).

**Table 2:** Parameters of linear models for the effects of sex, housing conditions, and diets and time on pre-hibernation mean minimum body temperature of juvenile garden dormice

Response variable	Term	Estimate ±SD	t value	*p* value
Mean minimum body temperature (°C)	Sex	0.87 ± 0.45	1.94	0.06
	Housing conditions	−3.27 ± 0.53	−6.12	**<0.001**
	Diet	−2.11 ± 0.46	−4.53	**<0.001**
	Time	−0.33 ± 0.09	−3.60	**<0.001**

Housing conditions were either single or grouped, and diets were *ad libitum* (AL) or intermittently fasted (IF). *p* values in bold indicate statistically significant effects. *p* values shown in bold correspond to statistically significant and interpretable values.

### Food intake and energy expenditure

**Figure 5:**
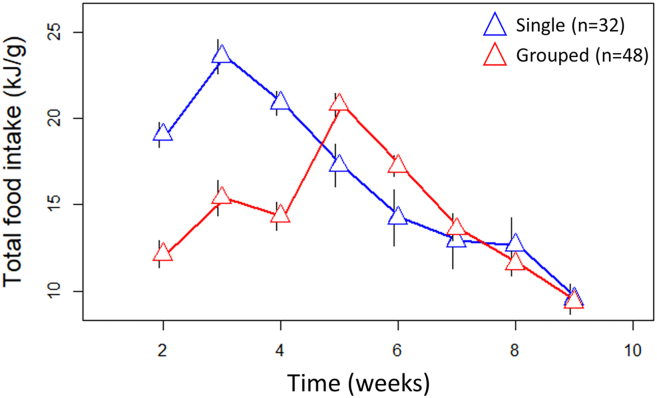
Food intake changes over time during the pre-hibernal period Total food intake (kJ/g) over time (weeks) between housing conditions (*n* = 48 grouped, *n* = 32 single) from the start of experiments until hibernation onset. Values are means ± SD.

Food intake was influenced by housing conditions and time (Table 3). Single individuals initially ate more and then decreased their food intake over time, whereas grouped individuals started with a lower food intake, increased it until a peak at the middle of the pre-hibernation period, and then progressively reduced their food intake until hibernation onset (Figure 5). Energy expenditure during pre-hibernation was influenced by housing conditions (Table 4). Grouped individuals show a lower total daily energy expenditure than single ones regardless of the diet (Table 5).

**Table 3:** Parameters of linear models for the effects of housing conditions (grouped, single), and time (week) on pre-hibernation food intake (KJ/g) of juvenile garden dormice

Response variable	Term	Estimate ±SD	t value	*p* value
Food intake (KJ/g)	Housing conditions	9.89 ± 1.69	5.85	**<0.001**
	Time	−0.24 ± 0.16	−1.56	0.12
	Group∗Time	−1.55 ± 0.29	−5.21	**<0.001**

Housing conditions were either single or grouped. *p* values shown in bold correspond to statistically significant and interpretable values.

**Table 4:** Parameters of linear models for the effects of housing conditions, body mass, and the diet on total daily energy expenditure and water turnover of juvenile garden dormice

Response variable	Term	Estimate ±SD	t value	*p* value
Total energy expenditure (KJ/d)	Housing conditions	12.32 ± 2.68	4.58	**<0.001**
	FFM	1.07 ± 0.27	3.86	**0.0003**
Water turnover (g/d)	FFM	0.18 ± 0.07	2.60	**0.012**

Housing conditions were either single or grouped, diets were *ad libitum* (AL) or intermittently fasted (IF). Fat-free mass was also assessed as an explanatory variable in the models. *p* values shown in bold correspond to statistically significant and interpretable values.

**Table 5:** Means and standard deviations for pre-hibernal total energy expenditure and water turnover of the experimental animal groups, according to housing conditions and diet

Response variable	Sex	Group	*n*	Mean ± SD
Total energy expenditure (KJ/d)	Male	Grouped AL	8	60.88 ± 7.47^a^
		Grouped IF	8	60.78 ± 6.43^a^
		Single AL	6	73.75 ± 6.15^b^
		Single IF	6	69.79 ± 27.42^b^
	Female	Grouped AL	8	55.31 ± 6.85^a^
		Grouped IF	7	51.58 ± 8.68^a^
		Single AL	6	71.13 ± 8.08^b^
		Single IF	6	66.48 ± 2.59^b^
Water turnover (g/d)	Male	Grouped AL	8	13.29 ± 1.56^c^
		Grouped IF	8	12.89 ± 2.05^c^
		Single AL	6	14.63 ± 2.42^c^
		Single IF	6	12.77 ± 5.04^c^
	Female	Grouped AL	8	11.15 ± 1.33^c^
		Grouped IF	7	11.82 ± 2.94^c^
		Single AL	6	12.23 ± 2.19^c^
		Single IF	6	12.41 ± 2.59^c^

Housing conditions were either single or grouped, diet was *ad libitum* (AL) or intermittently fasted (IF). Groups differing significantly in Tukey’s post-hoc comparisons are denoted by different superscripts.

### Growth, body mass gain and body composition

**Figure 6:**
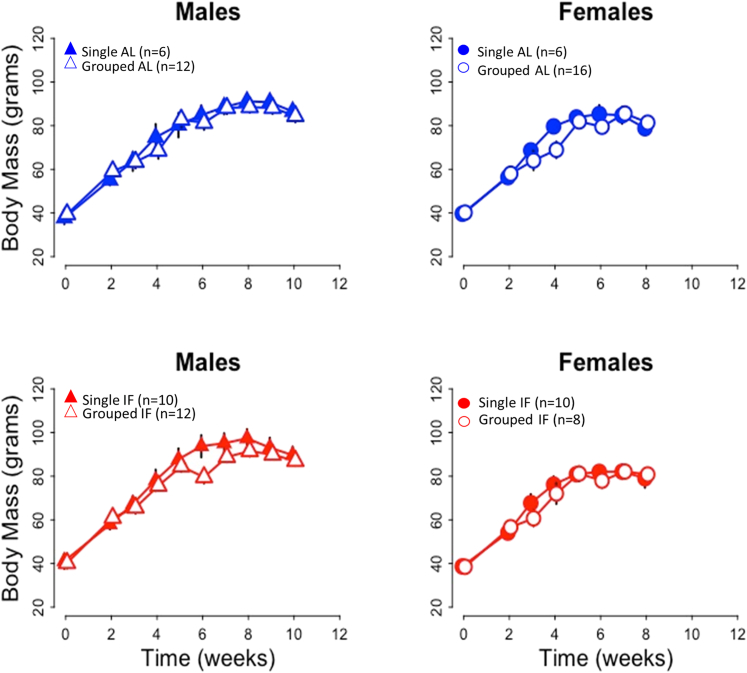
Body mass changes over time during the pre-hibernal period Change of body mass (grams) over time (weeks) between males (*n* = 40) and females (*n* = 40) in different diet conditions (*ad libitum* = AL *n* = 40, 40 fasted *n* = 40) and housing conditions (grouped *n* = 48, single *n* = 32) from the start of experiments until hibernation onset. Values are means ± SD.

Until the onset of hibernation, animals gained body mass over time until they reached a plateau. However, females seemed to reach a lower body mass, though non-significant, than males (Figure 6, and Tables 6 and 7). The absolute body length increased over time, and males reached a higher absolute body length than females (Table 7). Prior to hibernation, all the individuals showed similar fat mass levels, but males had a higher fat free mass than females, indicating that body mass growth was similar regardless of the diet and housing conditions (Tables 6 and 7).

**Table 6:** Parameters of linear models for the effects of time, housing conditions, and sex on pre-hibernal body mass, body length, fat mass, and fat-free mass of juvenile garden dormice

Response variable	Term	Estimate ±SD	t value	*p* value
Body mass (g)	Sex Time	2.48 ± 1.55 5.49 ± 0.14	1.61 40.31	0.11 **<0.001**
Body length (cm)	Sex Time	0.24 ± 0.07 0.15 ± 0.01	3.23 13.57	**0.001** **<0.001**
FM (g)	Group	0.64 ± 1.52	0.42	0.67
FFM (g)	Sex	5.57 ± 1.04	5.34	**<0.001**

Housing conditions were either single or grouped. *p* values shown in bold correspond to statistically significant and interpretable values.

**Table 7:** Means and standard deviations for pre-hibernal body mass, fat mass, and fat-free mass of the experimental animal groups, according to the sex, housing conditions, and diet

Response variable	Sex	Group	*N*	Mean ± SD
Body mass (g)	Male	Grouped AL	12	84.43 ± 8.07^a^
		Grouped IF	12	87.01 ± 6.27^a^
		Single AL	6	86.50 ± 4.98^a^
		Single IF	10	89.67 ± 9.45^a^
	Female	Grouped AL	16	81.37 ± 7.10^a^
		Grouped IF	8	80.81 ± 5.98^a^
		Single AL	6	78.10 ± 6.14^a^
		Single IF	10	79.08 ± 10.71^a^
Body length (cm)	Male	Grouped AL	12	12.37 ± 0.77^a^
		Grouped IF	12	12.11 ± 0.65^a^
		Single AL	6	12.20 ± 0.82^a^
		Single IF	10	12.15 ± 0.68^a^
	Female	Grouped AL	16	12.11 ± 0.79^b^
		Grouped IF	8	11.95 ± 0.55^b^
		Single AL	6	12.08 ± 0.83^b^
		Single IF	10	12.21 ± 0.44^b^
FM (g)	Male	Grouped AL	8	35.51 ± 4.96^a^
		Grouped IF	8	38.36 ± 4.61^a^
		Single AL	6	38.31 ± 5.65^a^
		Single IF	6	41.69 ± 6.18^a^
	Female	Grouped AL	8	39.54 ± 5.44^a^
		Grouped IF	7	38.49 ± 4.92^a^
		Single AL	6	37.18 ± 7.79^a^
		Single IF	6	35.49 ± 5.03^a^
FFM (g)	Male	Grouped AL	8	46.10 ± 4.01^a^
		Grouped IF	8	49.46 ± 10.48^a^
		Single AL	6	49.87 ± 3.01^a^
		Single IF	6	49.78 ± 7.97^a^
	Female	Grouped AL	8	43.02 ± 3.05^b^
		Grouped IF	7	45.96 ± 6.76^b^
		Single AL	6	44.60 ± 6.07^b^
		Single IF	6	42.24 ± 4.09^b^

Housing conditions were either single or grouped, diets were *ad libitum* (AL) or intermittently fasted (IF). Results are presented as mean ± SD. Groups differing significantly in Tukey’s post-hoc comparisons are denoted by different superscripts.

### Winter hibernation

#### Hibernating patterns

Females hibernated for sixteen weeks and males for fourteen weeks during winter. Both sexes emerged from hibernation at the same time, in mid-March (12^th^-15^th^), according to our colony of garden dormice kept under natural fluctuations of temperature and photoperiod.^21^ Total time spent in euthermia (total IBE duration) was significantly influenced by the hibernation duration as well as the interaction of sex, diet and hibernation period (early vs. late hibernation, Table 8). Especially in the first part of hibernation, the diet effect was stronger in males than in females. Individuals fed AL had a higher total arousal duration than fasted dormice. This effect was stronger in males than in females and was more pronounced during the first part of hibernation. Moreover, garden dormice stayed longer in arousal in the second part of hibernation than in the first part of hibernation. Also, within the same sex and within the same part of hibernation, AL-fed individuals had a higher arousal frequency than fasted individuals. This effect of diet was stronger in males than in females and especially in the second part of hibernation (Table 8). Males fed AL had a high arousal frequency compared to males fasted and all the females. Arousal frequency was also higher in the first part of hibernation compared to the second part (Figure S2 and Table 9). Finally, mean torpor duration was higher in the second part of hibernation than in the first part and higher in fasted dormice than in AL-fed individuals. Especially, males fed AL had the lowest mean torpor duration compared to the other experimental groups, especially during the second part of hibernation (Figure S2 and Table 9).

**Table 8:** Parameters of linear models for the effects of sex, diet, and hibernation period on hibernation torpor parameters of juvenile garden dormice

Response variable	Term	Estimate ±SD	t value	*p* value
Total IBE duration (h)	Sex	−6.01 ± 7.57	−0.79	0.43
	Diet	−31.05 ± 7.18	−4.32	**<0.001**
	Period	25.86 ± 9.39	2.76	**0.007**
	Winter duration	0.09 ± 0.01	7.28	**<0.001**
	SexF∗ Period1∗AL	−34.37 ± 14.91	−2.31	**0.02**
	SexM∗Period1∗AL	21.24 ± 9.62	2.21	**0.03**
	SexF∗Period1∗IF	−23.37 ± 11.49	−2.03	**0.04**
	SexF∗Period2∗AL	−21.80 ± 10.47	−2.08	**0.04**
Arousal frequency	Sex	−0.34 ± 0.55	−0.61	0.54
	Diet	−3.24 ± 0.57	−5.69	**<0.001**
	Period	−2.87 ± 0.44	−6.49	**<0.001**
	Winter duration	0.002 ± 0.001	6.18	**<0.001**
	SexF∗ Period1∗AL	−0.36 ± 0.98	−0.36	0.72
	SexM∗ Period1∗AL	−0.17 ± 0.53	−0.33	0.74
	SexF∗ Period1∗IF	0.92 ± 0.57	1.61	0.11
	SexF∗ Period2∗AL	−2.37 ± 0.81	−2.93	**0.005**
Mean torpor duration (h)	Sex	−23.50 ± 15.19	−1.54	0.13
	Diet	28.45 ± 16.05	1.77	0.08
	Period	77.40 ± 9.97	7.77	**<0.001**
	Sex∗diet	25.03 ± 21.72	1.15	0.26
	Sex∗ Period	−42.77 ± 13.87	−3.08	**0.003**
	Diet∗ Period	8.85 ± 14.35	0.62	0.545
	Sex∗ Period ∗diet	53.39 ± 19.80	2.69	**0.009**

Diets were *ad libitum* (AL) or intermittently fasted (IF), and hibernation periods were early (period 1) or late (period 2). Hibernation length was included in the models. *p* values shown in bold correspond to statistically significant and interpretable values.

**Table 9:** Hibernating parameters according to sex, diet and hibernation period

Response variable	Hibernation period	Group	*n*	Mean ± SD
Total IBE duration (h)	Early	Female AL	22	24.7 ± 19.5^ac^
		Female IF	18	4.60 ± 19.6^a^
		Male AL	18	74.3 ± 23.4^bd^
		Male IF	22	21.9 ± 5.4^ae^
	Late	Female AL	22	63.1 ± 21.4^b^
		Female IF	18	53.9 ± 4.3^bc^
		Male AL	18	78.9 ± 28.1^d^
		Male IF	22	47.9 ± 12.1^be^
Arousal frequency	Early	Female AL	22	11.4 ± 1.27^a^
		Female IF	18	9.6 ± 1.1^b^
		Male AL	18	10.5 ± 1.7 ^ab^
		Male IF	22	7.5 ± 1.1^c^
	Late	Female AL	22	4.6 ± 1.3^d^
		Female IF	18	3.6 ± 0.9^d^
		Male AL	18	6.5 ± 1.9^c^
		Male IF	22	3.3 ± 0.38^d^
Mean torpor duration (h)	Early	Female AL	22	151.8 ± 11.5^ab^
		Female IF	18	180.3 ± 20.1^a^
		Male AL	18	128.4 ± 23.4^b^
		Male IF	22	181.8 ± 20.1^a^
	Late	Female AL	22	229.3 ± 26.4^c^
		Female IF	18	266.6 ± 36.6^cd^
		Male AL	18	163.1 ± 34.2^a^
		Male IF	22	278.7 ± 32.9^d^

Diets were *ad libitum* (AL) or intermittently fasted (IF), and hibernation periods were early (period 1) or late (period 2). Results are presented as mean ± SD. Groups differing significantly in Tukey’s post-hoc comparisons are denoted by different superscripts.

#### Metabolic rate during hibernation

**Figure 7:**
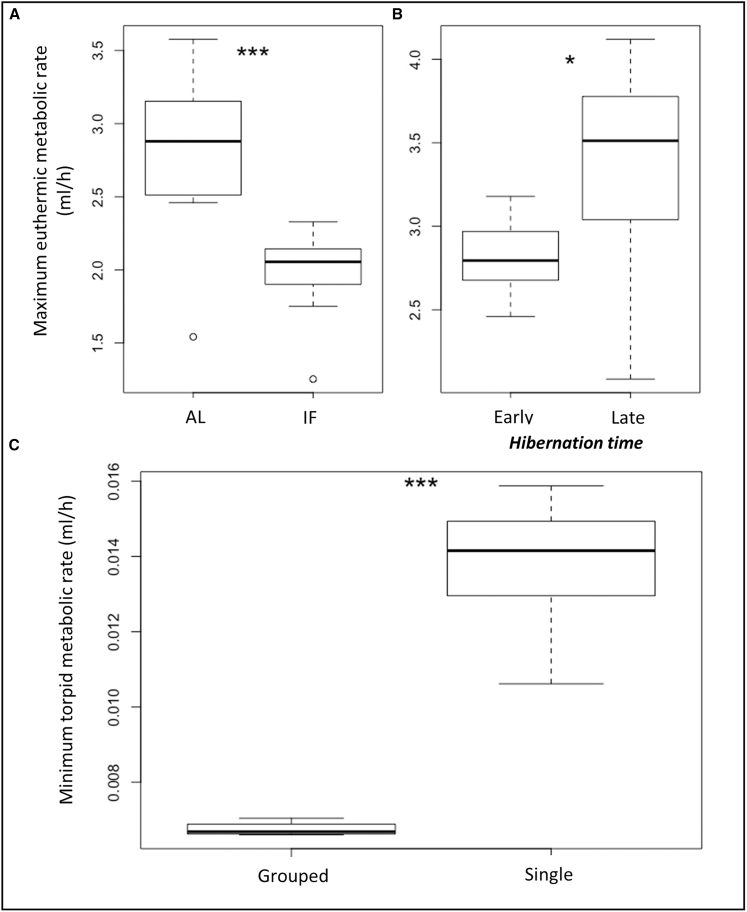
Changes in metabolism during the hibernation period Changes in metabolic rate (A, B, maximum euthermic metabolic rate: Max EMR, and C, minimum torpid metabolic rate: Min TMR) according to diet (AL = *ad libitum n* = 12, IF = fasted *n* = 12), hibernation period (early or late) and housing conditions (grouped, *n* = 16 or single, *n* = 8). Values are means ± SD. Significance was assessed using type II ANOVA (Wald χ^2^ tests) on linear mixed-effects models. Asterisks indicate significance levels (∗*p* < 0.05, ∗∗*p* < 0.01, ∗∗∗*p* < 0.001).

Animals hibernating with food showed a higher maximum euthermic metabolic rate (EMR) compared to fasted ones, and this parameter was higher in the second part of hibernation (Figures 7A and 7B, and Table 10). Additionally, during torpor, huddling animals reduced their metabolism compared to single individuals, as their minimum thermal response (minimum torpid metabolic rate, Min TMR) was lower (Figure 7C).

**Table 10:** Parameters of linear models for the effects of housing conditions, diet and hibernation period on maximum euthermic metabolic rate and minimum torpid metabolic rate of hibernating juvenile garden dormice

Response variable	Term	Estimate ±SD	t value	*p* value
Max EMR	Diet	−0.85 ± 0.21	−4.10	**<0.001**
	Period	0.54 ± 0.21	2.56	**0.02**
Min TMR	Housing conditions	0.007 ± 0.001	11.92	**<0.001**

Housing conditions were either single or grouped, diets were *ad libitum* (AL) or intermittently fasted (IF), and hibernation periods were early or late. Maximum euthermic metabolic rate: max EMR; minimum torpid metabolic rate: min TMR. *p* values shown in bold correspond to statistically significant and interpretable values.

#### Food intake, body mass, and body composition during and at emergence from hibernation

**Figure 8:**
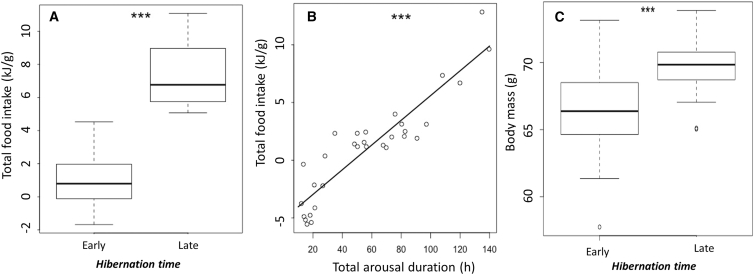
Food intake, body mass, and body composition during and at emergence from hibernation Total food intake and body mass changes over time. Total food intake is expressed in relation to time (early or late hibernation, A) and total arousal duration (B), as well as body mass during early and late hibernation (C). Values are means ± SD. *N* = 80. Significance was assessed using type II ANOVA (Wald χ^2^ tests) on linear mixed-effects models. Asterisks indicate significance levels (∗∗∗*p* < 0.001).

The total food intake was significantly higher in the second part of hibernation (Figure 8A and Table 11). Also, garden dormice stayed longer in arousal with increasing food intake (Figure 8B). Garden dormice lost body mass over hibernation regardless of diet conditions (Tables 12 and 13). However, the body mass loss in the late hibernation phase was higher than during early hibernation (Figure 8C).

**Table 11:** Parameters of linear models for the effects of hibernation period (early or late) and total arousal duration (IBE duration) on food intake of hibernating juvenile garden dormice

Response variable	Term	Estimate ±SD	t value	*p* value
Food intake (KJ/g)	Period	6.35 ± 1.23	5.18	**<0.001**
	Total IBE duration	0.11 ± 0.01	9.95	**<0.001**

Hibernation periods were early or late. Total arousal duration; total IBE duration. *p* values shown in bold correspond to statistically significant and interpretable values.

**Table 12:** Parameters of mixed linear models for the effects of housing conditions, diet, pre-hibernation body mass, hibernation period and winter duration on body mass, and effects of group, diet, pre-hibernation fat mass, fat-free mass on fat-mass and fat-free mass changes over winter of juvenile garden dormice

Response variable	Term	Estimate ±SD	t value	*p* value
Body mass (g)	Diet	0.72 ± 0.045	1.62	0.11
	Period	3.46 ± 0.69	5.04	**<0.001**
	Winter duration	−0.006 ± 0.008	−7.56	**<0.001**
	Pre-hib BM	0.86 ± 0.02	39.22	**<0.001**
FM (g)	Housing conditions	−2.78 ± 1.56	−1.78	0.11
	Diet	2.52 ± 1.55	1.63	0.12
	Winter duration	0.003 ± 0.004	−0.68	0.51
	Pre-hib FM	0.39 ± 0.12	3.22	**0.002**
FFM (g)	Winter duration	−0.001 ± 0.004	−0.26	0.79
	Pre-hib FFM	0.51 ± 0.17	2.93	**0.005**

Housing conditions were either single or grouped, diets were *ad libitum* (AL) or intermittently fasted (IF), and hibernation periods were early or late. Pre-hibernation body mass: Pre-hib BM; fat mass: FM; fat-free mass: FFM. *p* values shown in bold correspond to statistically significant and interpretable values.

**Table 13:** Means and standard deviations for post-hibernation body mass, fat mass and fat-free mass of the experimental animal groups, according to the housing conditions, diet and sex

Response variable	Sex	Group	*n*	Mean ± SD
Body mass (g)	Male	Grouped AL	12	63.79 ± 5.79^a^
		Grouped IF	12	69.44 ± 6.06^a^
		Single AL	6	65.83 ± 5.59^a^
		Single IF	10	69.16 ± 8.28^a^
	Female	Grouped AL	16	64.69 ± 5.95^a^
		Grouped IF	8	64.58 ± 4.34^a^
		Single AL	6	60.20 ± 6.99^a^
		Single IF	10	60.20 ± 8.36^a^
FM (g)	Male	Grouped AL	7	17.97 ± 4.16^a^
		Grouped IF	8	19.97 ± 5.48^a^
		Single AL	6	15.97 ± 5.83^a^
		Single IF	6	19.38 ± 7.41^a^
	Female	Grouped AL	8	21.67 ± 4.94^a^
		Grouped IF	5	19.32 ± 4.46^a^
		Single AL	6	15.07 ± 4.33^a^
		Single IF	5	17.96 ± 8.87^a^
FFM (g)	Male	Grouped AL	7	46.09 ± 4.01^a^
		Grouped IF	8	49.46 ± 10.48^a^
		Single AL	6	49.86 ± 3.01^a^
		Single IF	6	47.69 ± 6.84^a^
	Female	Grouped AL	8	43.02 ± 3.05^b^
		Grouped IF	5	44.57 ± 6.54^b^
		Single AL	6	45.13 ± 6.63^b^
		Single IF	5	42.24 ± 4.09^b^

Housing conditions were either single or grouped, diets were *ad libitum* (AL) or intermittently fasted (IF). Results are presented as mean ± SD. Groups differing significantly in Tukey’s post-hoc comparisons are denoted by different superscripts.

At emergence from hibernation, animals had the same body mass and body composition regardless of the diet conditions. Although sex was not included in the best model (Table 12), post-hibernation fat-free mass was higher in males than females (Table 13) which is consistent with higher pre-hibernation values.

#### Overwinter losses (body mass, fat mass, fat-free mass)

Garden dormice lost body mass over hibernation regardless of diet conditions (Tables 14 and 15). However, the losses of fat mass and fat-free mass differ according to housing conditions and the diet (Tables 14 and 15). Huddling individuals lost less fat mass than single individuals during hibernation. Huddling animals fed AL also lost more fat-free mass than huddling fasted individuals (Table 15).

**Table 14:** Parameters of linear models for the effects of housing conditions, diet and sex on body mass, fat mass and fat-free mass loss of juvenile garden dormice

Response variable	Term	Estimate ±SD	t value	*p* value
Body mass loss (%)	Housing conditions	−1.71 ± 0.92	−1.87	0.067
FM loss (%)	Housing conditions	−10.25 ± 3.48	−2.15	**0.004**
	Sex	1.16 ± 3.47	0.33	0.74
FFM (%)	Sex	−4.26 ± 3.17	−1.34	0.19

Housing conditions were either single or grouped, diets were *ad libitum* (AL) or intermittently fasted (IF). Fat mass loss: FM loss; fat-free mass loss: FFM loss. *p* values shown in bold correspond to statistically significant and interpretable values.

**Table 15:** Means and standard deviations for overwinter body mass, fat mass and fat-free mass losses of the experimental animal groups, according to the housing conditions, diet and sex

Response variable	Sex	Group	n	Mean ± SD
Body mass (%)	Male	Grouped AL	7	−22.78 ± 4.46^a^
		Grouped IF	8	−20.23 ± 2.42^a^
		Single AL	6	−23.92 ± 4.19^a^
		Single IF	6	−22.90 ± 3.55^a^
	Female	Grouped AL	8	−20.52 ± 2.14^a^
		Grouped IF	5	−22.67 ± 3.36^a^
		Single AL	6	−24.41 ± 4.17^a^
		Single IF	5	−21.82 ± 1.49^a^
FM (%)	Both	Grouped AL	15	−46.52 ± 9.89^a^
		Grouped IF	13	−44.22 ± 8.69^a^
		Single AL	12	−59.71 ± 10.27^b^
		Single IF	11	−51.43 ± 18.14^b^
FFM (%)	Both	Grouped AL	15	−8.85 ± 5.89^a^
		Grouped IF	13	−3.87 ± 8.48^a^
		Single AL	12	−1.12 ± 7.48^a^
		Single IF	11	−8.56 ± 12.53^a^

Housing conditions were either single or grouped, diets were *ad libitum* (AL) or intermittently fasted (IF). Fat mass: FM; fat-free mass: FFM. Results are presented as mean ± SD. Groups differing significantly in Tukey’s post-hoc comparisons are denoted by different superscripts.

## Discussion

The present study investigated the effects of huddling and food availability on the processes of growth and body mass gain, torpor use, metabolic rate, and energy intake in late-born juvenile garden dormice. We demonstrated that dormice prior to winter used different strategies of energy saving for reaching their necessary body mass and size. During pre-hibernation, juvenile dormice were either maximizing torpor use through metabolic depression in association with hypothermia or maximizing huddling behavior to reduce heat loss while maintaining stable euthermic T_b_. In contrast, during winter, no substantial effects of huddling were seen on hibernating patterns of juvenile dormice. Instead, we found that food availability, with a stronger effect in males than in females, influenced hibernation behavior. Fasted individuals maximized metabolic depression during winter hibernation, while AL-fed individuals stayed longer and more frequently in arousal. This flexibility of energy-saving strategies allows juveniles to effectively cope with a fluctuating environment that is likely to occur in times of unpredictable climatic events during global change.

### Contrasted energy-saving strategies of juvenile dormice during pre-hibernation

#### Huddling reduces torpor use prior to hibernation

When provided with food AL, huddling individuals showed lower torpor frequency than single individuals shortly before the hibernation onset. Huddling is known to minimize heat loss and to reduce energy expenditure by between 6% and 53%.^3^ If animals were IF, they all clearly increased their torpor frequency in the last phase of pre-hibernation in both housing conditions. This suggests that only the combination of high food availability and reduced heat loss through huddling in the group allowed animals to allocate energy into remaining a warm body and reduce torpor use, including its associated costs, like a higher level of oxidative stress, negative effects on immune competence,^22^^,^^23^ disturbance of proper heart function,^24^ or immobility and therefore vulnerability to predators.^25^ Also, regarding total and mean torpor length and mean minimum T_b_ during pre-hibernation, the diet and housing conditions had a significant influence. Huddling and AL fed individuals spent less time torpid than single and fasted individuals during the last phase of pre-hibernation. Fasted-induced torpor was also described before in juvenile garden dormice as a response to the acute energetic challenge, and it was suggested that dormice increase their use of torpor late in the season to support pre-hibernation fattening rather than growth,^15^^,^^22^^,^^26^ as a sufficient body mass is crucial for surviving winter.^14^ Similarly, food-storing eastern chipmunks (*Tamias striatus*) minimize torpor expression when food is supplemented.^27^ Furthermore, huddling and AL fed individuals used shallower torpor, that is, had a higher mean minimum T_b_ than single and fasted animals, which entered deeper torpor. This is in line with avoiding low T_b_ s and its associated risks^23^ and the incompatibility of low T_b_ during torpor with the high metabolic process of structural body growth.^17^ During the first weeks of pre-hibernation, torpor use was low in all groups. Different phases of pre-hibernation torpor use in late-born garden dormice were also reported in previous studies: a low torpor use during the intensive growth phase of the first 4 weeks and an increasing torpor use prior to the start of hibernation, after the termination of growth and when the body mass plateaued.^17^ Besides, female juveniles started to use torpor earlier, with a higher frequency of torpor and longer total torpor duration than male juveniles during pre-hibernation. Therefore, females entered winter hibernation two weeks earlier than males, as also described in previous studies.^28^ Interestingly, in the last three weeks of pre-hibernation, total torpor length in fasted males was higher than in males and females fed AL and fasted females. This could be due to a maximization of energy savings and allocation of energy into fattening rather than remaining a warm body and preparing for reproduction,^16^ as it has been previously demonstrated that IF juveniles reproduced less during the following year than those that were fed AL prior to hibernation.^17^

#### Huddling reduces food-intake prior to hibernation but does not affect growth trajectory and body mass gain

Housing conditions affected food intake in the pre-hibernal phase. Single dormice ate more food than huddling individuals. The energetic benefits of huddling have been reported previously, resulting in lower food intake, reduced water loss, and/or a more constant T_b_, along with a significant reduction in metabolic rate.^3^ Looking at the time courses of food intake during pre-hibernation, both single and huddling juveniles decreased their food intake progressively approaching winter, as they increased their torpor use throughout pre-hibernation. But while single individuals started the experiment with a high food intake in autumn, huddling individuals ate little in the beginning, increased their food intake until a peak mid pre-hibernation, before reducing their food intake again until the onset of hibernation. The low food intake in huddling juveniles in the beginning may be related to getting used to the new group constellation and the conspecifics, building a hierarchical structure^29^ and probably facing associated disturbances by conspecifics.^30^

In this study, no significant effect of huddling was found regarding body mass gain and growth during pre-hibernation in juvenile garden dormice, as these parameters did not differ between housing conditions. Single and fasted juveniles were able to gain body mass at the same rate and had the same growth level as huddling and individuals fed AL in the pre-hibernation. Consequently, all animals reached a similar body mass and body size at the start of hibernation. However, we found that huddling helped individuals to reduce their energy expenditure, with no consequences on body mass and growth. Prior to hibernation, both grouped and single dormice used torpor (Figure 2). We hypothesize that isolated individuals might have used less torpor than they would have to save energy, as grouped individuals do, or that rewarming in a group might be less energetically costly than for single individuals.

Moreover, the diet did not influence body mass, fat content, and body size either. Previous studies showed that intermittently fasting had no effect on growth rate, maximal pre-hibernation body size and fat content in late-born juvenile garden dormice.^15^^,^^17^ Studies in other hibernating species, such as arctic ground squirrels, have found similar results.^31^ Moreover, studies about social thermoregulation in rabbit pups (*Oryctolagus cuniculus*) showed that huddling further reduced the brown adipose tissue thermogenesis, which is also activated in torpor during the rewarming phases, by providing public warmth when pups are the most vulnerable, huddling buffers cold challenges, and ensures a constant allocation of energy to growth by delaying non-shivering thermogenesis.^11^ Contrary to these findings,^3^^,^^11^ this study did not support the aspect that huddling increases growth and fattening, as juvenile garden dormice reached a similar body mass and body size. As hibernators are seasonal species, they are strongly influenced by circannual rhythms that require a regulated body mass and body size at specific times of the year. Regardless of the circumstances, juveniles must prepare for hibernation to survive the winter and be able to reproduce in the subsequent period. Our results show that males have higher fat-free mass content and body length than females prior to hibernation, and this difference persists at emergence. As males must remain competitive upon emergence from hibernation, initiating hibernation with a higher fat-free mass content than females could be advantageous for the next reproductive season. These differences in fat-free mass and body length do not impact body mass, and both sexes exhibit a similar fat mass. However, females have a slightly lower body mass than males, which may reflect the difference in their fat-free mass.

### Food availability affects hibernation behavior of juvenile dormice during the winter

#### Males are affected more by food availability than females

Regarding winter hibernation, food availability affected the hibernation of juvenile garden dormice; however, males were particularly sensitive to the presence of food. Individuals fed AL had a higher total arousal duration and a higher torpor frequency than fasted dormice, but this effect was stronger in males than in females. This might be due to preparation for the reproductive season, as at emergence from hibernation, male garden dormice form groups with hierarchical structures, and the weakest or smallest animals have reduced access to females.^29^ An early occupation of good territories may be even more important for males than for females, as demonstrated in edible dormice.^32^ Especially late-born juveniles, as is the case in our study, may be encouraged to prepare for reproduction sooner. Accordingly, late-born individuals were found to breed at higher proportions than early-born animals during the following reproductive year.^17^ This suggests that being born late in the reproductive season is associated with a fast life history, that is, rapid growth, fast reproduction and short life span. Furthermore, previous studies suggest that endocrine function and tissue growth may be inhibited by the low T_b_ s associated with torpor, indicating that hormonal and growth processes are temperature-dependent in mammals.^16^ They suggest further that the testis growth in golden-mantled ground squirrels (*Spermophilus lateralis*) during the hibernation season was restricted to intervals of arousal from torpor. Young males could thus benefit from lower torpor use to ensure sexual maturation and future reproduction. Finally, our study showed that both sexes emerged at the same time from hibernation, while males in other species exhibit earlier emergence (the Gray Mouse Lemur, *Microcebus murinus*^33^). We also found that total arousal duration in both sexes and diet conditions was higher in the second phase of hibernation than in the first phase, although males fed AL spent the most time in arousal. Further studies will be needed to investigate the subsequent reproductive success of late-born juveniles in different diet and housing conditions, as well as long-term or transgenerational effects.

#### Food intake is higher in the late part of winter hibernation

During the hibernation phase, food intake was higher in the second part, as well as the total arousal length. Consequently, max EMR was higher in the second part of hibernation and in dormice hibernating with food compared to fasted individuals. It is known that the gastrointestinal tract is one of the most metabolically intense organs in terms of energy utilization and protein synthesis and is expensive to maintain.^34^ If the organ is not being used, it is reduced in size and activity.^35^ On the other hand, food-storing species that feed during interbout euthermia maintain a functional digestive system and intestinal absorption capacities during hibernation, optimizing nutrient assimilation during arousals from torpor,^36^ which leads to a higher metabolic rate. The higher food intake, less arousal frequency, and higher arousal duration in late hibernation compared to the early hibernation phase indicate a circannual effect due to an approaching emergence from hibernation. The garden dormice are thus beginning to prepare themselves for emergence and hence for the breeding and growing season. Garden dormice usually reach their adult size within the first two year^21^; the animals in the present study can be classified as juveniles or yearlings that must continue to grow to compete with more mature individuals.

#### Huddling does not affect overwintering body mass and fat-free mass losses, but influences fat mass loss

During winter, huddling had no substantial effect on hibernating patterns of juvenile dormice, except for a maximization of metabolic depression during torpor in huddled individuals. Despite this fact, no effect of huddling was found regarding body mass loss during winter hibernation in juvenile garden dormice, as these parameters did not differ between housing conditions. Single and fasted juveniles lost body mass at the same level during winter hibernation. Both groups lost more body mass in late hibernation than in early hibernation. Consequently, all juveniles reached a similar body mass at the emergence of hibernation in the spring. Huddling was reported to reduce heat dissipation, hence energy loss during rewarming in juvenile garden dormice, as most of the nestmates benefited from passive rewarming.^9^ Animals took turns in initiating arousals during winter; therefore, no energetic benefit of huddling was detected over the entire winter on an individual level. Just as in the pre-hibernal phase, body mass strongly underlies photoperiodic clues in hibernators. A sufficient body mass, especially for juveniles, should be the priority, regardless of the circumstances, as it is crucial for further survival and reproductive success.

Although all individuals showed the same body mass loss over winter, interestingly, huddling individuals lost less fat mass than those that were single. Huddled individuals showed a lower min TMR during hibernation, which could maximize energy savings, hence fat reserves during torpor.^37^ It remains to be determined in which way fat mass and fat-free mass utilization contribute to meet the energetic demand during torpor and rewarming in hibernating juvenile dormice and how huddling influences this contribution.

### Limitations of the study

Experiments presented in this article were conducted in captivity under constant environmental conditions including stable ambient temperature, i.e., conditions that do not capture the environmental variability experienced by juvenile dormice in nature. As a result, the expression and regulation of energy-saving strategies may differ, likely underestimated, under natural fluctuations in temperature, photoperiod, and resource availability where they may be even more pronounced. Further, huddling was addressed primarily as a thermoregulatory process, without accounting for its social dimension. In natural populations, group composition may influence huddling dynamics and individual energetic outcomes. These constraints should be considered when interpreting the results, and future studies are needed to assess their generality in more ecologically and socially realistic contexts.

To conclude, this study highlights the effects of huddling in combination with torpor, a behavior used by juvenile garden dormice in response to food availability. Dormice showed different strategies for reaching their necessary body mass and body size for surviving winter according to experimental conditions of housing and food availability. As expected, huddling behavior did influence torpor patterns and food intake during the first pre-hibernation period of an individual’s life. However, although juveniles used variable expressions of torpor modulated by huddling behavior, late-born juvenile garden dormice grew and fattened similarly during pre-hibernation and reached a similar body mass and body size at hibernation onset, as well as the emergence from hibernation. During winter hibernation, access to food significantly impacted hibernation parameters in males, likely due to preparations for the reproductive season, and this effect varies according to housing conditions. In the continuity of our study, it would be interesting to investigate the subsequent torpor patterns, reproductive behavior and success of late-born juvenile garden dormice, raised under different diets and housing conditions and to detect if the different energy saving strategies in their early life affect their somatic maintenance and future survival.

## Resource availability

### Lead contact

Requests for further information and resources should be directed to and will be fulfilled by the lead contact, Sylvain Giroud (sgiroud@nmu.edu).

### Materials availability

This study did not generate new unique reagents.

### Data and code availability

• All data are available in the Figshare public data at: https://doi.org/10.6084/m9.figshare.31429901.
• This study does not report original code.
• Any additional information required to reanalyze the data reported in this study is available from the lead contact upon request.

## Acknowledgments

This research was funded in most parts by the Austrian Science Fund (FWF; P31577-B25) to S.G. For open access purposes, the author has applied a CC BY public copyright license to any author acepted manuscript version arising from this submission. The funders had no role in study design, data collection and interpretation, or the decision to submit the work for publication. We thank P. Steiger for his valuable help with animal care. We also want to acknowledge A. Four-Chaboussant for her help with animal husbandry and care during the experiments. Additional thanks go to S. Smith for the English editing of the manuscript and to the anonymous reviewers for their comments that helped improve the quality of the work.

## Author contributions

S.G. and C.G. conceived and designed the study. J.E. and J.P.-G. performed surgeries for implantation of temperature loggers. A.Z. performed stable isotopes analyses; B.S. validated stable isotopes analyses. L.M.C. and S.G. performed the experiments, and L.M.C. and B.S. computed the data under the supervision of S.G. S.G.V., B.S., and L.M.C. performed the statistical analyses. L.M.C. and B.S. drafted the manuscript. S.G., A.B., and C.G. substantially edited and critically revised the manuscript. All coauthors commented on the manuscript and agreed on its content.

## Declaration of interests

The authors declare no competing interests.

## STAR★Methods

### Key resources table

**Table:** 

REAGENT or RESOURCE	SOURCE	IDENTIFIER
**Deposited data**		

All data are available in the Figshare public data	This paper	[Database]: [https://doi.org/10.6084/m9.figshare.31429901]

**Experimental models: Organisms/strains**		

*Eliomys quercinus* (Garden dormouse)	Breeding colony, Research Institute of Wildlife Ecology, University of Veterinary Medicine Vienna, Austria	BMBWF-68.205/0175-V/3b/2018

**Software and algorithms**		

R software	R Foundation	Version 4.0.3

**Other**		

Deuterium oxide 99.80% Microbiological T	Eurisotop	DLM-2259-1s
Servopro 4100 O2 and CO2 analyzer	Servomex	Servopro 4100
Mass flow meters FMA 3100	Omega Engineering	FMA 3100
Gas mixing pump type 55A27/7a	H. Wösthoff	55A27/7a
Dual-channel O_2_ analyzer	Sable System	Oxzilla OXZ-1401
Isotope ratio mass spectrometer (Delta V Plus)	Thermo Scientific	Delta V Plus
High-temperature conversion elemental analyzer (HTC-EA)	Thermo Scientific	HTC-EA
Conflo III interface system	Thermo Scientific	Conflo III
CTC PAL autosampler	CTC Analytics	CTC PAL
Temperature data logger	Research Institute of Wildlife Ecology (FIWI)	Customized temperature logger
Ketamine	Richter Pharma	Ketamidor 10%
Xylazine	Bayer	Rompun 2%
Isoflurane	CP Pharma	Isoflurane (1 mL/mL)
Meloxicam	Boeringer Ingelheim	Metacam
Suture material	SMI AG	Surgicryl PGA USP 3/0 and 4/0

### Experimental model and study participant details

#### Ethics statement

All experiments carried out in this study were approved by the Ethics and Animal Welfare Committee of the University of Veterinary Medicine, Vienna in accordance with the University’s guidelines for Good Scientific Practice and authorized by the Austrian Federal Ministry of Education, Science and Research (ref BMBWF-68.205/0175-V/3b/2018) in accordance with current legislation.

#### Animals

A total of eighty juvenile garden dormice (*Eliomys quercinus*) in their first year of life were investigated in 2018-19 and 2019-20. In 2018-19, fifty-six animals (28 females, 28 males) and in 2019-20 twenty-four animals (12 females, 12 males) entered the experiments. All individuals were born late in the reproductive season (07-20 Aug. 2018; 11-23 Aug. 2019). All animals were maintained under captive conditions at the Research Institute of Wildlife Ecology (FIWI) of Vetmeduni Vienna, Austria (latitude 48°15′ N, longitude 16°22′ E). For identification, all juveniles were individually marked with miniature subcutaneous transponders (Tierchip Dasmann, ANIMAL ID ISO 11784/85 FDX-B Standard, Tecklenburg, Germany). Animals were divided into two housing conditions and were kept either singly or in groups of four individuals of the same sex during the entire experimental time. During the pre-hibernal period (see below “Experimental design”), garden dormice were housed indoors in cages (60 × 40 × 40 cm) provided with branches and a self-made plastic nest-box (PVC tube; 110D x 17H cm) padded with hay and connected to the front door of the cage. Throughout that time, juveniles were held at stable ambient temperature (T_a_) of 20°C but exposed to natural variation of photoperiod. During winter hibernation (see Experimental design), animals were kept in standard plastic cages (36 × 20 × 14 cm), each connected to a plastic nest-box and placed in refrigerators under constant darkness and at a stable T_a_ of 4°C. Food was provided in the form of rodent pellets (ssniffHA, ssniff GmbH, Soest, Germany), which contained 19.0% protein, 3.3% fat, and 4.9% fibers, as well as with meal worms, exclusively during the pre-hibernal phase. Water was provided *ad libitum*.

### Method details

The experimental design is shown in Figure 1. Experiments were carried out on animals between September 2018 and April 2019 (24.09.2018–03.04.2019), and between September 2019 and April 2020 (24.09.2019–18.03.2020). After weaning at the age of six weeks with a body mass of 40.0 g ± 5.0 g, animals could habituate to the cages and nests for 2–3 days before entering experiments. Individuals were randomly separated into different experimental groups: fed *ad libitum* versus intermittently fasted, and huddling versus single individuals. The experimental groups consisted of 32 single individuals (16 males, 16 females) and 48 grouped “huddling” animals (24 males, 24 females). Grouped animals were placed in a cage as a group of four individuals of the same sex and of similar weight, with only one nest allowing them to build a huddle. Half of the animals (6 single males, 6 single females, 12 grouped males, 16 grouped females) were fed *ad libitum* (AL) during the entire experiment. The other half of the animals (10 single males, 10 single females, 12 grouped males, 8 grouped females) was intermittently fasted (IF), i.e., food restricted, during the same period by removing food every other day. To track individual torpor patterns, animals were implanted with small, customized temperature data loggers (FIWI, Vienna, Austria; resolution: 0.1°C, accuracy: ± 0.06°C) that monitored their core body temperature (T_b_) every 4 min. Loggers were removed and temperature data collected at the end of winter hibernation. Temperature in the nest was also continuously recorded through a customized nest temperature data logger (FIWI, Vienna, Austria; resolution: 0.2°C, accuracy: ± 0.06°C), with a connection outside the cage during the pre-hibernal phase, and outside the refrigerator during winter hibernation to collect data without disturbing the animals. These data were used to determine when animals were torpid^15^ during pre-hibernation and entering prolonged (>24h) torpor as sign of readiness for winter hibernation. We also used nest temperature recording for ethical reasons during the winter to ensure that all animals were properly hibernating in the refrigerators. However, T_b_ was used to determine individuals’ actual torpor and hibernating patterns (threshold of 18°C) for accuracy of data analyses. To assess metabolic rate (MR), respirometry measurements were recorded for 24 individuals (four single females, four single males, two groups of four males, and two groups of four females) during winter (see below for more details). To determine changes in body mass all individuals were weighed once a week during the pre-hibernal period, as well as at the start of hibernation, at mid-hibernation (early February) and post-hibernation, to the nearest of 0.1 g, using a balance (Mettler Toledo, PM34, Delta Range). To assess growth, the body-length of each garden dormouse was measured from the tip of the nose to the base of the tail using a measuring tape every two weeks during pre-hibernation, as well as at hibernation onset and post-hibernation. Animals remained in the pre-hibernal phase until they showed prolonged torpor (>24h) and a plateauing of body mass, i.e., ∼85 g for females and ∼90 g for males.^15^^,^^17^^,^^21^

#### Topor use and activity time

During the pre-hibernation period, we used measurements of nest temperature as a proxy for T_b_ to estimate torpor use and activity time. Core T_b_ recorded during winter hibernation was used to compute the total hibernation length, torpor frequency, mean torpor duration, and total time spent in euthermia (interbout arousal time = IBE duration).

#### Diet and food intake

Individual food intake was measured during cage cleaning by weighing the provided food and the remaining food and calculating the difference through subtraction. Food intake in grouped individuals was calculated by dividing the food intake of the entire group by four (number of individuals in the group). During winter hibernation, IF animals spent the hibernating period completely without food and water while AL individuals had *ad libitum* access to food and water, that was provided in the form of jelly-agar. For the agar cushions, 15 g of powder-formed Agar-Agar was mixed with 1.5 L of tap water, then boiled for ∼1 h to enable the optimal gel-structure. Next, the liquid agar-water mixture was filled into a large plastic bag and cooled (4 h at ∼3°C) until a solid gel structure was formed. The gel was cut into small pieces (5 × 3 cm) and stored in a refrigerator at 3°C. During the winter-hibernation period, food pellets were dried overnight (∼14 h) in an oven at 50°C before weighing. Food and water were refilled at regular intervals during cage cleaning. During the pre-hibernation phase, this procedure was done weekly, while in winter hibernation, cage cleaning was handled with special care every 3-4 weeks to ensure minimal disturbance for the torpid animals. Therefore, the refrigerators were opened in the completely darkened room, using red light for enabling visibility. The dirty cages were quickly exchanged with already equipped clean ones, so that the nests, where the animals were hibernating, remained untouched for minimal disturbances.

#### Determination of body composition, total energy expenditure and water turnover

In 2019–2020, body composition, total daily energy expenditure (TEE) and water turnover (rH_2_O) were assessed both at the onset and end of hibernation (*n* = 55) by the multi-point DLW methodology during a 6-day period.^15^^,^^17^ The body composition, i.e., fat-free mass (FFM) and fat mass (FM), was determined by isotopic dilution. A premixed 5 g/kg dose of DLW, thinned with 3% NaCl to physiological osmolarity, was injected intra-peritoneally into each animal. Isotopic equilibration in body water was determined by collecting a blood sample 1 h after the dose from the saphenous vein. The blood was collected in micro-capillary tubes, which were immediately flame-sealed. Pre-hibernal and post-hibernal FFM and FM levels, TEE, and rH_2_O were determined by the multi-point DLW methodology during a 6-day period, after the first 12 or 8 weeks of the pre-hibernal period, that is, at the plateauing of body mass, for AL and IF juveniles, respectively, or immediately at emergence from hibernation (post-hibernal). Analyses were performed by isotope ratio mass spectrometry at the M2iS platform of the Department of Ecology, Physiology and Ethology (CNRS IPHC UMR7178 Strasbourg, France), as described previously.^38^ The total body water was derived by the principle of isotopic dilution. FFM was calculated assuming a hydration coefficient of 73.2%, which has been reported to be very stable across species,^39^ and FM was computed by difference with body mass. Four individuals were excluded from the dataset due to anomalous body condition measurements.

#### Implantation and removal of temperature loggers

Surgical anesthesia was induced by an intramuscular injection of 50 mg/kg ketamine (Ketamidor10%, Richter Pharma, Wels, Austria) and 8 mg/kg xylazine (Rompun2%, Bayer, Leverkusen, Germany). Animals were maintained under gas relay using a flow of 1.5% isoflurane (1 mL/mL Isoflurane CP, CP Pharma, Burgdorf, Germany) in a 150 mL/min oxygen stream via facemask. For post-operative analgesia, 2 mg/kg Meloxicam (Metacam, Boehringer Ingelheim, Schweiz) was injected subcutaneously before surgery and repeated daily for the first 2–3 days. The operating field was prepared with Povidon-Iod and 2-Propanol, Ethanol 96% (Betaseptic, Mundipharma, Frankfurt, Germany) according to standard sterile surgical procedures and covered with sterile surgical drapes. Animals were placed in dorsal recumbency, and the abdominal cavity was opened through a 1 cm incision in the *linea alba* to introduce the temperature logger (1.7g) to the abdomen. Peritoneum and abdominal muscles were sutured using synthetic absorbable surgical suture material USP 3/0 (Surgicryl PGA, SMI AG, Hünningen, Belgium) using a single-button suture technique. In addition, synthetic absorbable surgical suture material USP 4/0 (Surgicryl PGA, SMI AG, Hünningen, Belgium) was used to suture the skin with a continuous intra-cutaneous suture technique. During the entire procedure, vital parameters: heart rate, respiration rate, and peripheral hemoglobin oxygen saturation as measured by pulse oximetry (SpO2) were monitored. After emerging from hibernation at the end of march, all loggers were removed from the animals undergoing the same surgical procedure.

#### Respirometry system and metabolic rate

To determine energy expenditure during winter, oxygen consumption (VO_2_) or MR was measured using an open-flow respirometry system.^40^ Six cages with twelve individuals were measured simultaneously, followed by the other six cages at a different time (two single females, two single males, four grouped males, and four grouped females, with half of them fasted and the other half fed *ad libitum*). Flow rates of approximately 40 L h^−1^ for single individuals and 80 L h^−1^ for groups were used and continuously measured using calibrated thermal mass flow meters (FMA 3100, Omega Engineering, Stamford, CT, USA). Oxygen concentration was determined by a dual-channel electrochemical O_2_ analyzer (Sable System, Las Vegas, USA). The analyzer was calibrated with nitrogen and air mixtures by a high-precision proportioning pump before and once during the experimental period. Air was pumped through the respiratory chamber with membrane pumps. All recordings were interfaced to a laptop. Relative humidity was measured in sampled air and used for correction within the calculations. Oxygen data were corrected for the analyzer’s drift by automatically switching to the sample reference air at regular intervals. VO_2_ was calculated using the following equation:

$$VO2=FD*\frac{FIO2-FEO2}{\left(1-FIO2*\;\left(1-0.85\right)\right)}\;\left(l/h\right)$$

(FD = dry flow, FIO_2_ = fractional concentration of O_2_ in the incoming airflow, FEO_2_ = fractional concentration of O_2_ in the outgoing airflow), assuming a respiratory quotient of 0.85.^41^

Once torpor and euthermia have been defined based on T_b_ profiles, MR data were presented separately as metabolic rate during torpor (TMR) and during interbout euthermia (EMR), more specific as mean torpid metabolic rate (mean TMR), minimum torpid metabolic rate (min TMR, calculated as the mean of the five lowest MR values) and maximum euthermic MR (max EMR, calculated as the mean of the five highest MR values).

### Quantification and statistical analysis

Statistical analyses were conducted using R (Version 4.0.3). To account for the lack of independence between individuals kept in the same group we included “Group ID” (group size of singly housed individuals equal to one) as a random factor in all statistical models described below for which data were available on individual level (i.e., all torpor parameters as well as body mass and body length but not food intake and metabolic rates, which could only be measured on group-level; see above). For all analyses during the pre-hibernal and hibernation periods, the normality of residuals from statistical models (see below) was assessed by inspecting quantile-quantile plots and histograms. When necessary, Box-Cox transformation was applied resulting in normally distributed residuals in all cases.

#### Pre-hibernal period

We tested the effects of housing condition and diet on pre-hibernation TEE and rH_2_O, and body composition by using linear model, also including body mass as an explanatory variable. Linear mixed-effects models (R-package lme4^42^) were used to test effects of sex, housing condition (housing), diet and their respective pairwise interactions with time (week) on body mass, relative body length, torpor frequency, total torpor length, mean torpor length, and mean minimum body temperature, respectively. Also, a linear mixed-effects model was used to test the effects of sex, housing condition and their respective pairwise interaction with time on relative food intake. Next to group ID (see above) year was included as a random effect, as data were collected in two different years, as well as “Animal ID”, which was nested within “Group ID”. We ran a model selection and kept the best model based on AICc (Akaike Information Criterion corrected for small sample size^43^^,^^44^ (R-package MuMIn^45^). We applied an ANOVA with type II sum of squares on the best model (R-package“car”^46^). We employed Tukey’s post-hoc tests (R-package “multcomp”^47^) to reveal specific differences when necessary. In figures, asterisks indicate significance levels for ANOVA results (∗*p* < 0.05; ∗∗*p* < 0.01; ∗∗∗*p* < 0.001), whereas different letters indicate significant pairwise differences revealed by Tukey’s post-hoc tests.

#### Winter hibernation - two periods of winter hibernation

To investigate body composition changes over winter hibernation, linear-mixed-effects models on post-hibernation fat mass and fat-free mass were used with Animal ID was included as a random factor, which additionally included the fixed effect of pre-hibernation fat mass and fat-free mass. The best model was kept after the model selection as described above. Finally, we also employed Tukey’s post-hoc tests (R-package “multcomp”^47^) to reveal specific differences when necessary.

Previous studies have reported seasonal changes in telomere dynamics during hibernation, including increases in relative telomere length (RTL) toward late hibernation in spring, suggesting the presence of a seasonal or circannual regulatory program in hibernators.^40^ In line with these findings and supported by preliminary analyses indicating temporal shifts in physiological parameters across winter, we divided the hibernation period into early and late phases (mid-February; week 9 for males and week 11 for females) to account for potential phase-specific effects. Therefore, for all explanatory variables in the models described below the respective pairwise interactive effects with hibernation period (i.e., early versus late hibernation) were added. Linear mixed-effects models were used to test effects of sex, housing condition, diet, and their interactions (including the three-way interaction) on torpor frequency, total time spent in euthermia (interbout arousal time = IBE duration), mean torpor duration. To correct for different hibernation durations among individuals, the respective length of each individual’s winter hibernation period was included as another fixed effect. To investigate body mass change over winter hibernation a linear-mixed-effects model on post-hibernation body mass was used that included additionally the fixed effect of pre-hibernation body mass. As these analyses included repeated measurements “Animal ID” nested within “Group ID” nested within year was used as random effect in models on torpor parameters and body mass, and “Group ID” nested within year in the model on food intake, respectively. In the model on food intake, only the year was included as the sole random effect, as data were only available on the group level. Food intake in this model was additionally corrected for average group mass (average of pre- and post-hibernation group mass) by division to account for different group sizes in singly and group-housed animals. For obvious reasons, this model on food intake was only run with data from *ad libitum* fed animals and groups, and diet, therefore, was not included as a fixed effect. In the models on metabolic rates (i.e., max EMR and mean TMR), only linear models without random effects were used as data were only measured in one year and on the group level. The best model was kept after the model selection as described above. We applied an ANOVA with type II sum of squares on the best model (R-package“car” (Fox & Weisberg, 2019). Finally, we also employed Tukey’s post-hoc tests (R-package “multcomp”^47^) to reveal specific differences between the two parts of hibernation, diets, and sexes on the described parameters, when necessary. In figures, asterisks indicate significance levels for ANOVA results (∗*p* < 0.05; ∗∗*p* < 0.01; ∗∗∗*p* < 0.001), whereas different letters indicate significant pairwise differences revealed by Tukey’s post-hoc tests.
