# Supplementary material for: Huddling and food availability shape seasonal torpor and energetics of juvenile garden dormice
Source: iScience. 2026 Jun 4;29(6):116211. doi: 10.1016/j.isci.2026.116211 (PMC13266128; doi:10.1016/j.isci.2026.116211)
Supplement: Document S1. Figures S1 and S2 [file mmc1.pdf]

iScience, Volume *29*

Supplemental information

Huddling and food availability shape seasonal torpor and energetics of juvenile garden dormice

Laura Magaly Charlanne, Beata Sente, Sebastian G. Vetter, Joy Einwaller, Johanna Painer-Gigler, Audrey Bergouignan, Alexandre Zahariev, Caroline Gilbert, and Sylvain Giroud

Supplemental material

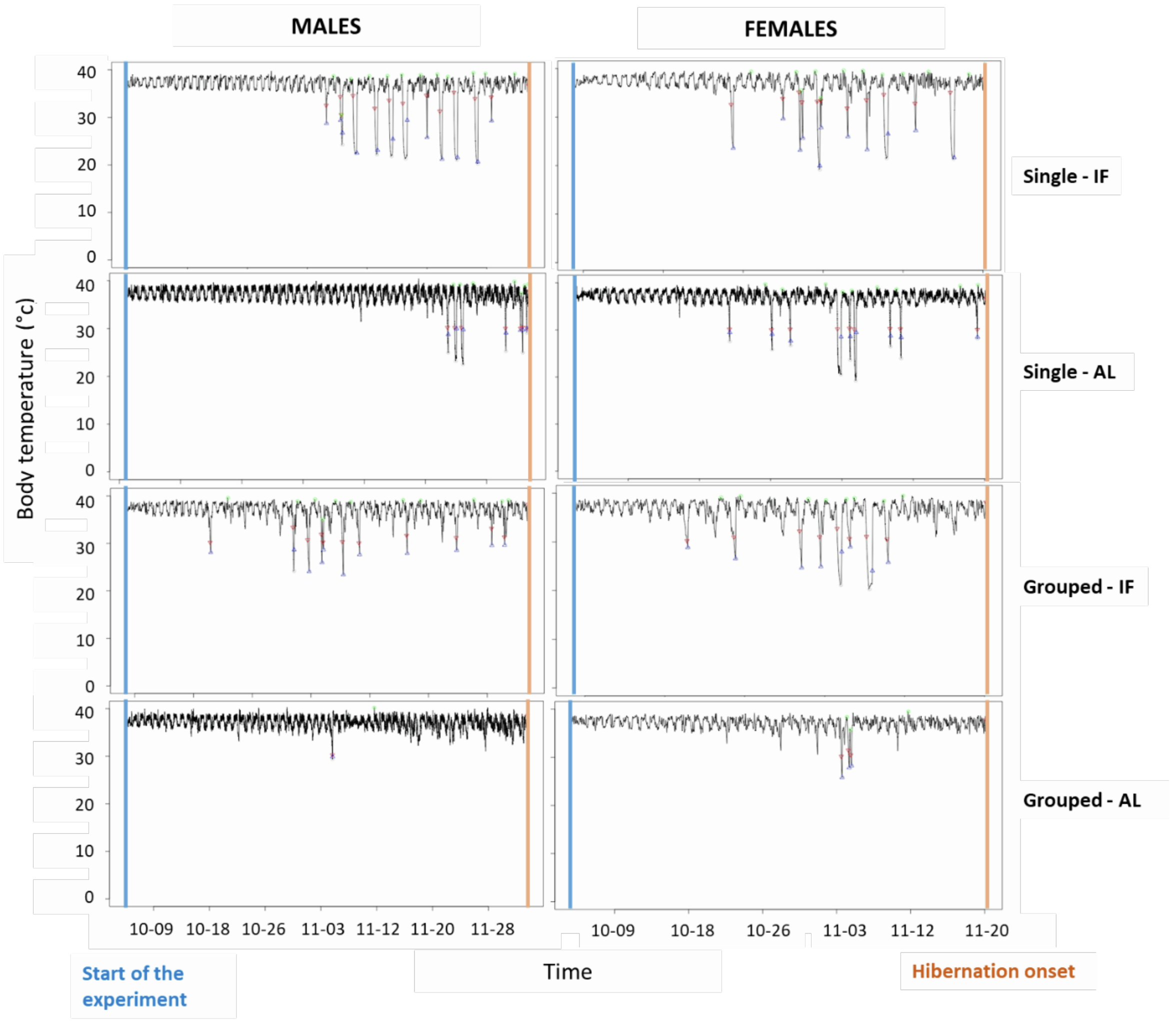

Figure S1. Representative individual body temperature profiles during the 2018 pre-hibernal period for each experimental group.

Groups are single-housed or grouped individuals, either fed *ad libitum* of intermittently fasted, separated by sex. Vertical lines show the start of the experiment (blue) and the onset of hibernation (orange).

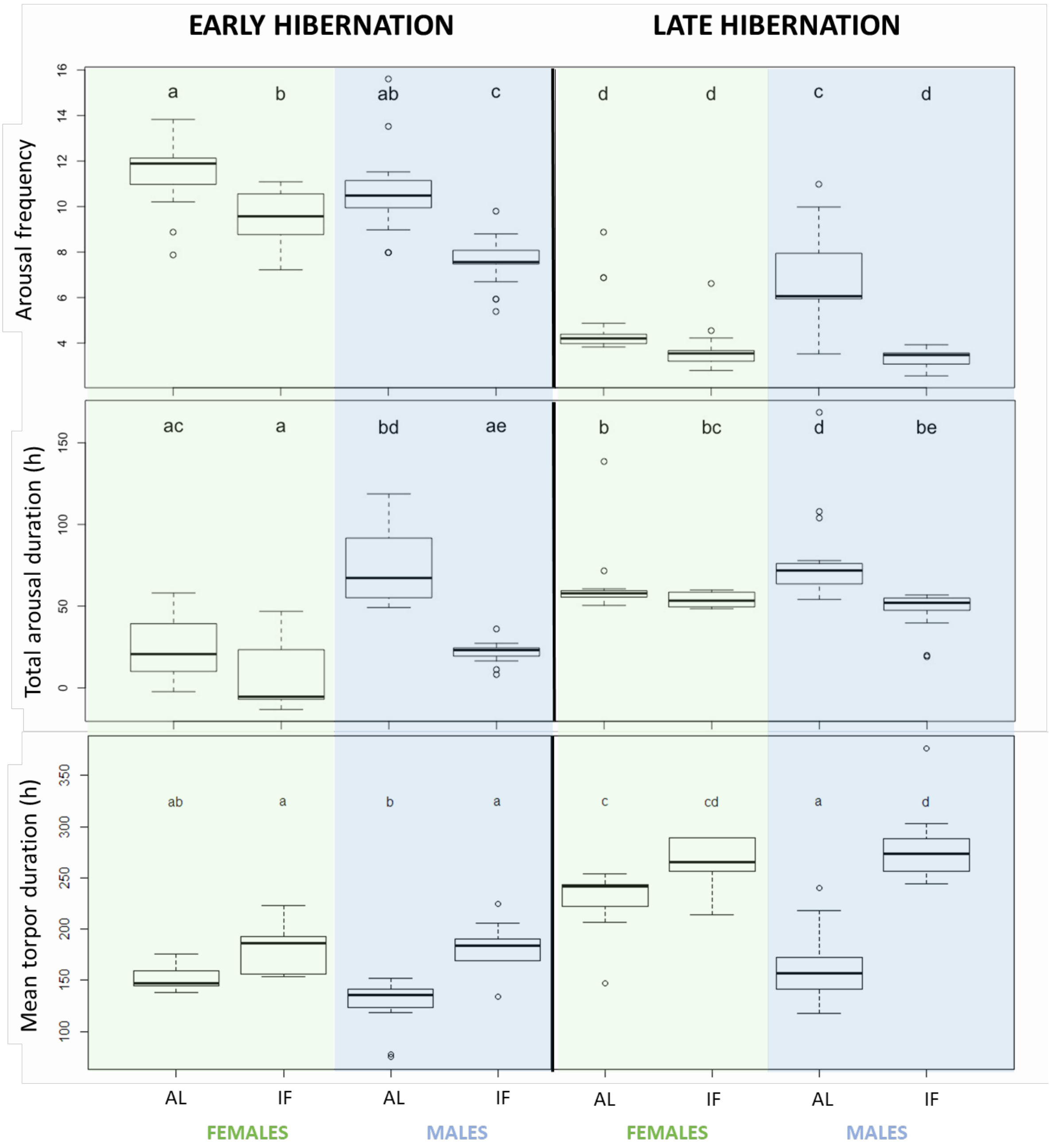

Figure S2. Hibernating parameters according to sex and diet (ad libitum 'AL' and intermittently fasted 'IF') and time (early and late hibernation).

Results are presented as mean ± SD. Groups differing significantly in Tukey's post-hoc comparisons are denoted by different superscripts.
